# Theorizing competition: An interdisciplinary framework

**DOI:** 10.1177/10245294251330343

**Published:** 2025-04-08

**Authors:** Carina Altreiter, Claudius Gräbner-Radkowitsch, Stephan Pühringer, Ana Rogojanu, Georg Wolfmayr

**Affiliations:** Institute for Sociology and Social Research, Vienna University of Economics and Business, Vienna, Austria; Institute for Sociology, Johannes Kepler University, Linz, Austria; Department of Pluralist Economics, 38982 Europa Universität Flensburg, Flensburg, Germany; Institute for the Comprehensive Analysis of the Economy, 27266 Johannes Kepler University, Linz, Austria; Institute for the Comprehensive Analysis of the Economy, 27266 Johannes Kepler University, Linz, Austria; Socio-Ecological Transformation Lab, Johannes Kepler University, Linz, Austria; Institute for Sociology and Social Research, 27254 Vienna University of Economics and Business, Vienna, Austria; 31294 FH Campus Wien, Vienna, Austria; Institute for Sociology and Social Research, 27254 Vienna University of Economics and Business, Vienna, Austria; Institute for the Comprehensive Analysis of the Economy, 27266 Johannes Kepler University, Linz, Austria

**Keywords:** Competition, interdisciplinarity, methodology of the social sciences and humanities, B41, B5, P16

## Abstract

This paper introduces a framework to facilitate an interdisciplinary analysis of “competition.” While such an interdisciplinary analysis can be justified by referencing the various fields of social and economic life in which “competition” is important, three challenges are found to aggravate such endeavor: First, a challenge of scope, which refers to the generality of the concept of “competition” and the fact that contributions differ with regard to the competing actors, the objects of competition, as well as the institutional specificities of competition; second, a challenge of methodology, which exists because different scientific disciplines study competition with distinct methods and epistemological orientations, and, third, a challenge of normativity, which refers to the fact that debates about competition have been closely linked to controversial political debates. To mitigate these challenges, and to explicate the often implicit meta-theoretical assumptions in the scope, methodological, and normative dimension, this paper introduces a meta-theoretical framework. Its usefulness is illustrated via a comparative description of selected contributions from the social sciences and humanities. Despite its limited scope, it yields some preliminary conjectures that may inspire future research: first, there are sufficient common elements across different concepts of competition that justify an interdisciplinary approach to study competition; second, apart from differences between disciplines, there are remarkable differences within disciplines that are at least of similar importance. Finally, there are important interdependencies between the meta-theoretical dimensions considered in the framework.

## Introduction

Competition has become central across economic, political, and social fields (Arora-Jonsson et al., 2021b; Hartmann and Kjaer, 2015; Stark, 2020a). Its influence extends beyond economic coordination to relationships between nation-states (Kapeller et al., 2019; Werron, 2012), policy design (Kjaer, 2015), academics (Musselin, 2018; Wetzel, 2013), arts and entertainment (Tauschek, 2012), and even romantic relationships (Wetzel, 2013). Some scholars trace back its relevance to its role as a characteristic feature of neoliberalism (Foucault, 2010), its role in the economization of the social (Jessop, 2012; Schimank and Volkmann, 2012), or it being an example of the performativity of economics and economic ideas (Callon, 1998; Mackenzie et al., 2008; Pühringer, 2018). Others argue that competition has already been a fundamental principle of social organization much earlier (Nullmeier, 2002; Simmel, 1995 [1903]; Werron, 2015). This ubiquity makes competition an attractive subject of investigation for various disciplines (Gane, 2019; Werron, 2015).^1^

Much current research happens within disciplinary boundaries, employing different conceptualizations, perspectives, or methodologies to examine competition.^2^ In some cases, such disciplinary approach is adequate, yet the assertion of an increasing relevance of competition in various social fields also suggests an *interdisciplinary* approach is warranted in other cases. For instance, does competitization represent an expansion of *economic* principles or does competition formats in fields like education, arts, or entertainment present *distinctive* phenomena that differ from economic competition? Are there common properties of these wide arrays of situations that warrant an interdisciplinary investigation? Or would such an interdisciplinary view rather shallow distinctive features of phenomena that required their own, more specific theoretical accounts? While a unifying and interdisciplinary approach can help to answer such questions that refer to different social contexts, it gets aggravated by the differences between disciplines, such as their distinct vocabulary, methodological approaches, and “codes of scientific conduct” (e.g., Gräbner and Strunk 2020).

The idea that an interdisciplinary approach to competition can be scientifically warranted has been articulated by, for example, Werron (2015) and Gane (2019), who investigate different disciplines since the 18th century, focusing on the normative dimension, or Jessop (2015), who examines differences in the understanding of competition in the economic and political field. These contributions have chosen a particular aspect of competition—mostly its normative assessment—for which they provide an interdisciplinary analysis. Arora-Jonsson et al. (2021a) echo the present paper’s vantage point, noting that “the idea of competition is currently found in many, if not most, social domains” (Arora-Jonsson et al., 2021a: 4), but lamenting “a piecemeal and disparate theorization across disciplines, a situation that discourages deeper questions and leaves little hope for constructive dialogue” (Arora-Jonsson et al., 2021a: 5). They delineate an umbrella definition that puts the subjective perceptions of the actors involved at center stage and facilitates the analysis of how competition is socially constructed.

Our approach is different but complementary: rather than strengthening theoretical foundations of competition research with an overarching definition, our endeavor is meta-theoretical: our main contribution is delineating a framework to facilitate a cross-disciplinary conversation on and analysis of competition.^3^ This framework provides blueprints for the systematic description of competition, thereby helping to understand commonalities and differences in existing works, as well as in various forms of competition in different fields of social life. Thereby, it addresses key challenges in interdisciplinary (or pluralist) research, as identified by previous research in philosophy, the social sciences, and educational psychology: (i) the lack of a shared vocabulary, (ii) the lack of a shared mental and substantial model of the subject of investigation, as well as (iii) meta-theoretical inconsistency (e.g., Claus and Wiese, 2019; Gräbner and Strunk, 2020; Sun et al., 2020). Or, more generally, “interdisciplinary teams need to elaborate and negotiate their common ground that is the basis of mutual knowledge and beliefs” (Claus and Wiese 2019: 192). Our framework helps them to do so by providing an external vehicle in the form of blueprints and guiding questions to develop this common ground. Notably, using blueprints and guiding questions in this context is a common strategy, as discussed in the literature on collective learning (see, e.g., Sun et al., 2020: 3–5 and the references therein).

The framework serves both theoretical and empirical purposes. Theoretically, it helps clarifying commonalities and differences between seemingly disparate conceptualizations of competition across disciplines. This way it facilitates a shared understanding that is a necessary pre-condition for triangulating concepts from different disciplines. Empirically, it can serve two different functions: first, it facilitates the choice, justification, and adjustment of the underlying theories of competition for the case at hand. Second, it helps mapping conceptions of competition originating from practical experiences of the people (or institutions) whose actions one investigates to academic concepts of competition.^4^

The paper proceeds in four steps: (1) diagnosing three *challenges of interdisciplinary competition research* (CICR); (2) addressing these challenges by introducing a meta-theoretical framework for triangulating different concepts of competition; (3) applying this framework to a selection of theoretical approaches; and (4) using the framework to derive conclusions regarding commonalities and differences in theoretical approaches to competition and sketching out ways to apply the framework empirically. The final Section concludes by discussing potentials and limits of the framework and suggesting avenues for further research.

## Challenges of interdisciplinary competition research

We begin by introducing our general working definition of competition. This definition is meant as a *minimal definition*: every constellation meeting all its requirements should be considered a competition. Thus, the definition potentially embraces a wide range of theoretical concepts and empirical cases, which may differ, for example, in whether competition and its components are seen as naturally emerging in specific contexts or as socially constructed (Arora-Jonsson et al., 2020), or whether competition is seen as a process, a relation, or an event (Arora-Jonsson et al., 2021a; Stark, 2020b). We understand competition as a constellation in which (i) at least two actors try to acquire (ii) a scarce (and rival, material or immaterial) good, and in which (iii) their interaction is structured by social institutions such that the allocation of the good depends on the actor’s relative performance according to one or more performance measures. This definition is meant to delineate *social competition*. For *natural* competition, the third requirement is not necessary. If one wished to focus on *economic* competition, one could add a fourth requirement: that the institutions structuring the interaction constitute a *market* on which prices can be formed (see Gräbner and Pühringer, 2021 for a more extensive discussion).

We found three dimensions to be of particular relevance when analyzing differences and commonalities across different disciplines. First, there are varieties in who the actors of competition may be, in what is competed for, and in the institutional context of competition. Second, approaches differ regarding their *epistemological* assumptions and the *methodological* tools, ranging from models for predicting the effects of competition to qualitative methods aiming to understand the effects on subjective self-understanding of competing individuals. Finally, different concepts of competition are also associated with different normative assessments.

Since the triangulation of different approaches to study competition across disciplines would require a shared understanding and a common ground regarding (meta-)theoretical assumptions, the just described variety aggravates such triangulation. More precisely, this paper addresses three key challenges to any interdisciplinary approach to competition:

1. The *challenge of scope*: the concept of “competition” is very general and contributions differ with regard to (i) the competing *actors* (“competition *among whom*?”), (ii) the *object* of competition (“competition *for what*?”), as well as the (iii) institutional specificities, such as the performance measures used (“competition *through what*?”).
2. The *challenge of methodology*: different disciplines study competition with distinct methods and epistemological orientations (i.e., receipts for how knowledge can be gained and requirements an adequate analysis must meet).
3. The *challenge of normativity*: debates about competition have been closely linked to controversial political debates. Some academics were and are actively involved, while others claim to present descriptive accounts.

We speak of “challenges’ because the diversity of approaches in terms of scope, methodology, and normativity across disciplines aggravates their triangulation. We believe these challenges concretize general challenges for interdisciplinary research—primarily the construction of a shared understanding of problems, methodologies, and solutions (e.g., Sun et al., 2020)—for the case of competition research. The proposed framework addresses these challenges by providing blueprints and guiding questions that facilitate the explication of often implicit assumptions on *scope*, *methodology*, and *normativity* and, thereby, help to establish the common ground of understanding that is necessary to make the joint investigation successful. In the literature on interdisciplinarity, pluralism, and, more generally, collaborative problem solving, the lack of shared rules and knowledge about research process originating from disciplinary practice, and a lack of explicitness regarding the assumptions on research objects, questions, methodologies, and goals have been argued to be main sources of problems (e.g., Claus and Wiese, 2019; Gräbner and Strunk, 2020; Sun et al., 2020). The extent to which these challenges also exist within single disciplines and whether our framework is useful in these contexts as well will be taken up in the last two Sections.

## An analytical framework for tracing the intellectual history of competition

Our analytical framework that addresses the CICR comprises three blueprints for the classification of concepts of competition according to their *scope*, *methodology*, and *normative* connotations. Each blueprint contains guiding questions as well as ideal types that facilitate the analysis of conceptions found in the literature or during fieldwork (see Tables 1–5). Unlike Weber’s “ideal types,” which stand for a condensed, abstract concept, we use the term to describe the most definite positions in each dimension, that is, to concretize concepts of competition from the literature which we consider to be good examples of extreme positions in the spectrum of the relevant categories. By pointing to these most extreme positions, we aim to illustrate the relevance of considering the differences along the dimensions. We do not suggest that all existing work can be classified within the ideal types. Rather, we expect much of the existing research to lie somewhere in between them.

**Table 1.** Analytical framework addressing the challenge of scope by explicating the competing actors.

Challenge of scope I—competing actors			
Guiding question I	Who is competing?		
Ideal types for GQ 1	Competition among individuals	Competition among groups	Competition among states
	Individuals compete with each other for the object of competition.	Groups compete with each other for the object of competition.	Nation states compete for the object of competition.
Examples	In a beauty contest, individuals compete against each other for the prize (Tauschek, 2013).	In political science, democracy is discussed as a form of competition between parties for the voters’ favor (Schumpeter, 1994).	Member states of the EU compete for the settlement of firms within a “European race for the best location” (Kapeller et al., 2019).

**Table 2.** Analytical framework addressing the challenge of scope by explicating the object of competition.

Challenge of scope II—object of competition		
Guiding question II	What is competed for?	
Ideal types for GQ 2	Particular	Universal
	Competition is considered to operate only in particular areas and its functioning is context-dependent. That means the competitors compete only for a well-defined subset of goods within a given field.	Competition is ubiquitous and always follows the same logic in different social fields. That means, there is a ubiquitous competition for a variety of goods in numerous social fields.
Examples	While classical economists studied competition for commodities, some sociologists and anthropologists such as Markus Tauschek focused on competition for social goods, for example, in the form of beauty or music contests.	For Pierre Bourdieu, competition is a universal principle operating in all social fields.

**Table 3.** Analytical framework addressing the challenge of scope by explicating the institutional framework of competition.

Challenge of scope III—institutional framework		
Guiding question III	How is the relative performance of the competitors assessed and linked to the distribution of the scarce good?	
Ideal types for GQ 3 I	Centralized allocation	Decentralized allocation
	There is a central entity that assesses the relative performances of the competitors and allocates the scarce good accordingly.	The assessment of the relative performances of the competitors, as well as the distribution of the scarce good, happens without a centralized entity.
Examples	Organized contests, such as sporting contests or architectural competitions, where judges award prizes after evaluating the contributions according to predefined rules (Stark, 2020a; 2020b).	In global financial markets, assets are traded among many suppliers and possible customers, with prices forming according to supply and demand.
Ideal types for GQ 3 II	Formalized criteria	Non-formalized criteria
	The criteria for assessing the relative performance of the competitors are formalized.	The criteria for assessing the relative performance of the competitors remain informal.
Examples	In architectural competitions for the allocation of building grounds and subsidies, there is clearly a defined set of criteria that includes architectural as well as ecological, financial, and social aspects (Azevedo et al., 2024).	On competitive markets for consumption goods, commodities are allocated to customers usually via their willingness to pay, which itself is the result of their preferences for prices and quality, none of which is directly observable.

**Table 4.** Analytical framework addressing the challenge of methodology.

Challenge of methodology			
Guiding question I	What kinds of methods are suggested for the study of competition?		
Ideal types for GQ 1	Model-based analysis	Abstract-direct analysis	
	Scholars represent their target system and competition through a model, analyze the model, and make conclusions based on the model analysis.	Scholars describe competition in their target system and base their conclusions on this description without relying on a model.	
Examples	In many economic paradigms, one regularly sets up a mathematical model, proves theorems, and relates these results to the target system.	Much of the current work in the humanities, social and cultural studies describes the behavior of people and derives more abstract claims from this description directly without building models.	
Guiding question II	What kind of explanations does the concept aspire?		
Ideal types for GQ 2	Hermeneutic explanations	Causal explanations	Functional explanations
	The goal of the analysis is to understand the role competition plays for the decision making of the actors involved.	The goal is to identify causal mechanisms.	The goal is to identify the function competition is playing.
Examples	A grounded theory approach to competition in housing would start by interviewing or observing people searching for housing to identify how competition affects them.	In many parts of evolutionary economics, scholars are interested to identify the root cause for the effect of competition.	Hayek explains the emergence of a competitive market economy via its superior role as an economic coordination device.

**Table 5.** Analytical framework addressing the challenge of normativity.

Challenge of normativity			
Guiding question I	Does the concept imply a normative connotation of competition?		
Ideal types for GQ 1	Descriptive intention	Prescriptive intention	
	The concept rests upon descriptions and comes without normative implications for policy and behavior.	The concept implies certain policies and institutions in reality, which would, if implemented, have beneficial or undesirable societal implications.	
Examples	The classical General Equilibrium Models were—before the introduction of the welfare theorems—meant as purely descriptive means to study competition between economic actors.	Adam Smith considers competition as a characteristic of his preferred system of natural liberty, which is why the term is positively connotated.	
Guiding question II	If the concept is normative, how is competition evaluated?		
Ideal types for GQ 2	Negative evaluation	Ambiguous	Positive evaluation
Examples	Wetzel (2013) criticizes the role of competition as a comprehensive governing principle of human conduct in neoliberal contexts.	Rosa (2006) points to positive as well as destructive consequences of competition.	For Hayek (1969), competition “discovers facts that would otherwise remain unknown” and is essential for economic success.

Regarding the *challenge of scope* one may classify concepts of competition according to (1) the competing actors, (2) what these actors are competing for, and (3) how their relative performance is measured and the allocation of the object of competition is institutionalized. Regarding actors (see Table 1), some concepts, particularly in anthropology, focus on competition among individuals, whereas others examine competition between higher-level entities like groups (e.g., parties in competitive theories of democracy (Schumpeter, 1994 [1943]) or nation states in the discussion about the “race to the best location” (e.g., Kapeller et al., 2019). Competition may appear simultaneously and in interdependent ways. For instance, rules that policy makers implement on the national level to ensure the competitiveness of their country might foster competition among individuals on the labor market. Moreover, one might even observe a complex nesting between various realms of competition and cooperation: the competitiveness of a firm in a market depends, for example, on how it has institutionalized the competition between its employees for promotions—but also on how it ensures a sufficient level of cooperation among them. And even when one remains on the level of firms as such, the same two firms can be both competitors as well as cooperators in different arenas of interaction—a phenomenon termed “coopetition” (see, e.g., Bengtsson and Kock, 2014).

In all these cases, the mechanisms operating on each level are likely to be different and the concrete set of actors, their relations, and their environments should be explicated when comparing different concepts of competition. Moreover, while the examples in the table can be identified as either a micro or a macro perspective, many scholars consider their concepts of competition to be applicable on different levels. And finally, the ideal types should not suggest a misleading homogeneity within these categories: “groups,” for instance, can refer to sets of individuals whose interactions are institutionalized very differently, for example, in the context of a profit-oriented firm, a political party, an educational institution, or a sports club (Arora-Jonsson et al., 2021c; Hasse and Krücken, 2013; Wolfmayr, 2024). Also, the ideal types should not suggest the absence of intermediate cases. They give examples corresponding to the common distinction between “micro,” “meso,” and “macro” levels, but in common multi-level frameworks, such as systemism, intermediate cases such as a “nano” level beneath the micro or a “mega” level above the macro might exist. For a more nuanced discussion of layered ontologies, see, for example, Gräbner and Kapeller (2017), and for practical examples see the Section on exemplary applications below.

Regarding to what the actors are competing for (see Table 2), the operation of competition might differ considerably, depending on whether competitors compete for material or immaterial, commodified or non-commodified goods. Some concepts, termed *particular* concepts, treat these different kinds of competition as different phenomena and, therefore, focus on competition in singular fields (e.g., economic competition for commodities or social competition for status). *Universal* concepts rather consider competition to be a universal logic, underlying dynamics in basically all social fields. Concrete applications are often located in the continuum spanned by these extreme positions.

Coming to the institutional setting of competition (see Table 3), a key question refers to the measures for the performance of the competitors and the mechanisms linking this performance to the allocation of the scarce good. This link may be implicit and unformalized: firms are assessed by customers based on the prices and quality they can offer. This coordination of supply and demand happens in a decentralized way, and preferences for low prices and high quality might exist only in the heads of the customers. Conversely, when architects compete for building ground in cities such as Vienna, they participate in an architectural competition according to a clearly defined and formalized set of criteria (Azevedo et al., 2024).

One might also focus on the institutions governing competition more generally. In sports, for instance, particular rules govern the competition among competitors, usually monitored by a referee. Changes in the rules might change the competences and means available to the referee. Economic competition among firms is also governed by rules. Here, the referee might be an organization such as the Bureau of Competition in the United States, and changes in the competition policy might change task and competences of such organizations.

The blueprint summarized in Table 4 addresses the *challenge of methodology*, concerned with the methods (e.g., statistics, dynamical systems modeling, computer simulation, participant observation, and interviews) and the epistemological orientation (regarding what is accepted as an adequate explanation) of different accounts on competition. In a first step, one should ask whether a concept explicitly or implicitly suggests a particular methodology for its analysis. To facilitate such meta-theoretical comparisons, the set of ideal types for the first guiding question draws on Weisberg’s (2007) distinction between *model-based* and *abstract-direct* analyses. In the first case, competition is investigated by first creating an artificial surrogate for the actual system under investigation—a model. This model is then analyzed and, in a third step, related to the system under investigation to derive statements about the latter. A prominent example is game theory.^5^ The model-based approach is typical for many schools in economics but less common in, for instance, anthropology: here scholars do not develop models that they investigate *instead* of their actual target system but rather focus on a *direct* analysis of the target. Weisberg calls this strategy *abstract-direct* analysis.^6^

The second guiding question aims to explicate the epistemology of a given concept of competition by asking how it means to derive new knowledge about the competition investigated. The ideal types refer to Elster’s (1983) different modes of explanations: explanations explicating the motives of the parties involved (“hermeneutic explanations”), explanations geared towards the explication of causal mechanisms (“causal explanations”), and explanations identifying the function a certain process (“functional explanations”). Hermeneutic explanations are often the goal of anthropological studies, functional explanations have been prominent in parts of Austrian (e.g., Hayek, 1969) or institutional economics (c.f. Wilber and Harrison, 1978), and causal explanations are regularly aspired by evolutionary economists (c.f. Witt, 2014). Notably, however, functional explanations have lost much of their appeal. At the same time, *evolutionary* explanations, that is, explanations that rest on the Darwinian paradigm that is now underlying many modern sciences, have become more popular. They explain phenomena by explicating the process of how they emerge from the Darwinian key mechanism of evolutionary selection. Whether they represent their own category can be debated. According to Witt (2014), for instance, a key feature of the Darwinian paradigm is a subscription to causal explanation (see also Hodgson, 2004: 8), so evolutionary explanations should be considered a (particularly prominent) subset of causal explanation.

Many disagreements in applied work can be traced back to differences on the epistemological level since the latter dictates the quality standards used, and the kind of explanation aspired dictates the research methodology used. Unfortunately, such meta-theoretical assumptions often remain implicit. This can prevent a constructive conversation across disciplinary boundaries since scholars from different disciplines have different quality criteria for the analysis of competition, design their studies to achieve different kinds of explanations, and, therefore, can have difficulties to understand and appreciate the work of others. Addressing this problem requires the explication of these different epistemological strategies because only then they can become a potential subject of constructive exchange. Thus, by explicating these differences, the present framework makes it possible to identify the ultimate sources of differences between concepts, thus paving the way for interdisciplinary triangulation (Gräbner and Strunk, 2020).

Finally, addressing the *challenge of normativity* requires explicating the academic, political, and socio-historical context of the concepts (see Table 5). We distinguish two ideal types, again spanning a continuum: *descriptive* concepts, meant to *describe* competition; and *prescriptive* concepts that im- or explicitly suggest certain avenues of reform. For instance, Hayek (1969) used his theory of competition as a discovery process to reject the attempt of Lange (1936), who advocated a socialist planning board based on his work. Neither of the two concepts can be properly understood without explicating the political debate in which they were developed. Once implicit value judgments are clarified, concepts with a prescriptive intention can be classified according to how positive or negative their view of competition is. It is, however, important to note that positions may not be explicit in their normative assessment, making their classification difficult. Equally, there has been a growing variety in normative assessments across the disciplines. Recognizing the normative implications of different concepts and their socio-historical and ideological embeddedness is nevertheless important, considering the migration of competition across social fields and contexts and the interrelation between academic concepts of competition and the practical implementation of competition. In practice, the proliferation of competition, as well as criticisms of competition and attempts to restrict it, is often inspired by scholarly debates (Ergen and Kohl, 2022). Thus, when empirically studying competition in a specific context, one may encounter normative assessments associated with concepts of competition arising from academic debates grounded in a particular normative framework.

## Exemplary applications of the analytical framework

This Section applies the analytical framework introduced previously to selected contributions in the literature. Because we need to focus on selected disciplines, the scope of this example is necessarily limited (see also the introduction and outlook). Its purpose is to demonstrate how the framework can be used on a theoretical level to build a solid basis for empirical work. The applications below demonstrate how mapping various contributions onto the framework’s common blueprint can aid comprehending similarities and differences between seemingly unrelated conceptualizations from different disciplines. This can promote a shared understanding across disciplines and can aid in comparing concepts and selecting theoretical accounts of competition for empirical analysis, as will be illustrated in the Section that follows.

### Scope of competition

#### The actors of competition

First theoretical concepts dealing explicitly with competition emerged during the 18th century in the field of Political Economy (comprising much of what today is separated into economics, philosophy, political science, and sociology). They were mainly concerned with competition *among firms and/or individuals*. Yet, despite the differences in wording, macroeconomic theories of the time also referred to competition, but only at the level of nation-states mercantilism, for instance, was characterized by an agonist understanding of international trade, suggesting countries keep most of the value-creating activities within their own boundaries. Until today, theories of competition in economics (and political science^7^) remain concerned with competition among individuals and/or firms, but competition among nations is now more explicitly recognized.

In social and cultural studies, the emphasis on theories of competition among individuals is more pronounced. However, as in economics, the variety of levels on which competition has been discussed has been increasing: Simmel’s influential conceptualization of competition as an indirect fight in which the distribution of a scarce good between two or more parties is managed by a third party was concerned mainly with individuals. Contemporary sociological or anthropological work extends this by studying love relationships and sports (Wetzel, 2013) and competitive formats such as beauty or music contests (Tauschek, 2012). Historians such as Jessen (2014) and Hölkeskamp (2014) or sociologists such as Rosa (2006) and Werron (2014a) moreover highlight the concept’s applicability across various analytical levels, from the everyday life of individuals to competition among organizations or nation states (e.g., Arora-Jonsson et al., 2021b).

#### The object of competition

Regarding the question of what players compete for, the scope of research has expanded: initially, it focused on competition for commodities, later concepts also considered non-commodified goods, such as *prestige*, *recognition,* or *attention*, while still focusing on one area of application. The late 20th century brought forth *universal* concepts of competition, which claim the applicability of the same concept to all fields of social life.

Classical economic theorists focused on commodified goods. Mill, for instance, was keen about restricting the scope of his theories of competition to the *economic realm*, where the distribution of material goods was of central importance. Similarly, in *substantivist economic anthropology*, scholars like Karl Polanyi, George Dalton, and Paul Bohannan studied how different societies organize their economies to satisfy material needs, focusing on competition for the distribution of the resources involved (Polanyi, 1977: 12). Early contributions to *economic sociology*, for example, Max Weber’s work Eeconomy and Society” (1978 [1922]), had a similar focus but Weber already anticipated a broader scope when referring to competition as a “peaceful attempt to attain control over opportunities and advantages which are also desired by others” (Weber, 1978 [1922]: 38). Despite his focus on economic activities, he also pointed to other social contexts in which competition may occur.

Georg Simmel, a contemporary of Weber, redirected competition research toward *social goods* in contexts like trade, love relationships, and sports. *Formal economic anthropologists* like Cyril Belshaw, Richard Salisburg, Fredrik Barth, and Harold K. Schneider took a different route, studying non-Western societies through a neoclassical economic lens. Consequently, also intangible social goods such as recognition, honor, respect, love, and prestige were understood as possible objects of competition. Today, scholars examine competition in different fields of social life and consider a wide range of scarce goods that might be at stake (Arora-Jonsson et al., 2021b). Rather than pursuing a general theory of competition, however, they tend to emphasize the uniqueness of competition in different contexts (Nullmeier, 2002; Tauschek, 2012, 2013).

This is different for concepts of *universal competition*, as popularized by scholars such as Gary Becker in economics and Pierre Bourdieu in sociology. In the 1970s Gary Becker (and his fellow economists George Stigler and James Buchanan), who considered competition as a universal coordination mechanism, applied economic methodology to issues such as crime, family, discrimination, marriage, death penalty, and human capital, all of which were considered to be characterized by the same principle of competition. Although using a different methodology, Pierre Bourdieu likewise argued that every social field is characterized by an economic, universal principle: in each field, there is competition for profits and the specific capital of the field (Bourdieu, 1992: 17). This meant that all fields, including cultural production (Bourdieu, 1993), the academic field (Bourdieu, 1992), or even a sexual field (Green, 2008; Illouz, 2012: 51–58), were characterized by a competitive rationality.

#### The mode of allocation

We suggest two guiding questions to structure approaches to competition when it comes to the mode of allocation: first, the degree of centralization of the allocation and, second, the degree of formalization of the performance measure.

Cultural scholars, like Markus Tauschek, often focus on *centralized* competition, such as beauty (2012) or music contests (2014) as “cultural performances” (Tauschek, 2012: 109) where specific actors (jurors, competitors, and audiences) interact according to specific cultural rules in a clearly delimited context. While centralized forms are less common in social and economic sciences, they still hold relevance, as evidenced, for instance, by research on the role of rankings and ranking institutions for the government structures in science (e.g., Brankovic et al., 2018), or the allocation of third-party funding to researchers (e.g., Schweiger et al., 2024). Here, the focus is on how centralized entities assess scientific work, how they allocate resources, what determines their acceptance, and what the implications of competitors’ strategies are for the scientific system more broadly.

The study of *decentralized* competition is especially significant in social sciences, particularly economics. The most famous example is the role of markets in economics, that is, institutions that help coordinating supply and demand for scarce goods via price mechanisms. Such decentralized forms of competition are sometimes also addressed by sociologists as evidenced, for instance, by Tobias Werron’s contribution on forms of competition in which the “third party” is not so clearly identified and remains largely anonymous (Werron, 2015: 200).

Regarding the performance criteria for allocating the scarce good, there are cases in which these are clearly explicated and formalized, such as in architectural competitions for the allocation of building lots and subsidies, where projects are assessed on architectural, economic, ecological, and social grounds (Azevedo et al., 2024). However, in Bourdieu’s and Simmel’s work, the criteria for distributing social recognition or emotional attention may remain implicit. One has to note, however, that as Hodgson (2004) stated, the distinction between “formal” and “informal” is not always clear. This is evidenced, for instance, by the case of hiring committees for professorships, which use highly formalized guidelines but are heavily influenced by informal criteria, like networks, sympathy, and strategic interests.

### Methodological dimensions

#### What kinds of methods for the study of competition are suggested?

Regarding methodological orientations, Weisberg (2007) distinguished *model-based* and *abstract-direct* analyses. There is a growing interest in *models* of competition, reflecting the growth of “model-based science” across disciplines (e.g., Magnani and Bertolotti, 2017). The archetype of a model-based approach to competition is the theory of *perfect competition* in economics, as developed by mathematicians such as Gerald Debreu (Debreu, 1991; Weintraub, 2002). By assuming utility- and profit-maximizing agents, and via the concept of economic equilibrium, scholars developed mathematical models that could be studied analytically. The epistemological focus was more on deriving mathematical results for the sphere of the models, rather than on the correctness of assumptions: “an axiomatized theory has a mathematical form that is completely separated from its economic content” (Debreu, 1986: 1265).

The absence of a pre-defined target system for these models, especially for the mathematical concept of (perfect) competition, facilitated the application to many different topics, such as “marriage markets,” “friendship markets,” or even “life markets”. This also applies to many critiques of this economic mainstream, like Shaikh (2016), who criticizes the neoclassical approach. Yet, his alternative approach of “real competition” also follows a model-based approach, only with a different formalism. While this “mathematization” of economics (Debreu, 1991) is a development of the 20th century, already classical economists such as John Stuart Mill viewed economics as an abstract science with an a priori method (Mill, 2000 [1844]), in which political economists must make assumptions to draw deductive conclusions.^8^

The situation is different in other social sciences and humanities, where both model-based and abstract-direct analyses are practiced, but with the latter being more prominent. Here, Simmel’s theory of competition as an indirect fight stands out methodologically. He examined concrete examples from everyday life, but his primary interest lay not in their meticulous description. Rather, it was in the abstraction from them and the deduction of logical consequences, such as the fact that opponents do not expend their energy to harm one another, but only to outperform each other (Simmel, 1995 [1903]: 224). He then took these logical conclusions and applied them to other social fields without having studied them empirically. This places him somehow in between the two ideal cases of Weisberg (2007). Nevertheless, most contemporary approaches in social and cultural studies prefer empirical approaches that can be classified as *abstract-direct representation*, often focusing on specific empirical case studies and emphasizing their unique features (Hölkeskamp, 2014; Tauschek, 2012). In summary, today both model-based and abstract-direct approaches to competition exist, yet different paradigms—and not disciplines as a whole—tend to exhibit a strong preference for one of them.

#### What kind of explanations does the concept aim for?

Another way to distinguish the methodological approaches to competition is by the *kind of explanation* aspired. This distinction matters as different kinds of explanations come with different evaluation standards. Much debate among scholars from distinct disciplines can be traced back to these differences. For example, most approaches to study competition in cultural studies seek what Elster (1983) classified as *intentional* (or “hermeneutical”) *explanations*. Here, the goal is to describe what actors hoped to achieve through their behavior, like presenting themselves favorably to enhance their chances to win a competition. Explanations may also describe the formation of intentions or beliefs about the implications of their actions (e.g., Tauschek, 2014). Since people’s reasons and desires are usually unobservable, scholars rely on methods such as interviews or participant observations to develop such intentional explanations. Many specific paradigms within the social studies (such as analytical sociology) and economics (especially in some parts of evolutionary economics, e.g., Witt, 2014) instead pursue *causal explanations*. These attempt to clarify the causal factors that have led to the emergence of certain forms of competition, such as competing for the favor of an anonymous audience (Werron, 2014a); that determine the mechanisms through which competition aggregates individual preferences; or that explain how different institutions influence the distribution of the goods actors are competing for.

Finally, *functional explanations* explain a phenomenon by highlighting the function it fulfills. They are less prominent today, but the new transaction cost literature, for instance, explains the emergence of social organizations via their functionality in facilitating transactions among individuals. Similarly, Hayek (1985) used functionalist reasoning when arguing that competitive markets have emerged because they represent “a more efficient allocation of societal resources than any design could achieve” (p. 63–64).

In some cases, distinguishing between different kinds of explanations can be challenging. Nevertheless, there is a tendency of certain disciplines—and paradigms within these disciplines—to prefer one type of explanation. As in the case of distinguishing model-based and abstract-direct explanation, differences between paradigms *within* disciplines appear to be more informative than differences *between* disciplines as a whole.

### Normative connotations and historical context

#### Does the concept imply a normative connotation of competition?

As indicated above, distinguishing descriptive and prescriptive concepts of competition is illuminating, yet also difficult since work that has clear normative implications often presents itself as being totally descriptive (e.g., Erhard and Jensen, 2017). Especially in economics, numerous studies of competition claim to be purely descriptive, a trend going back to classical economics when the distinction was important to many. John Stuart Mill, for instance, used the concept of competition in a supposedly descriptive analysis of the process of price formation in the sphere of production, while being explicit about being normative and prescriptive in his analysis of the sphere of distribution. Similarly, Leon Walras differentiated between economics as *pure science*, *applied economics* as a more practical approach, and *social economics* concerned with justice and ethics, with only the latter being explicitly prescriptive (Walras, 2003 [1874]). However, normative considerations were also at the foundation of his studies of “pure economics,” through which he tried to reconcile liberal and socialist economic ideas (Koppl, 1995).

In other disciplines, the motivation to delineate purely descriptive theories of competition is often different and more methodological: many contemporary conceptualizations of competition in the humanities, for instance, aspire *intentional* or *hermeneutic* explanations, meaning that theorists must not take a clear stance on competition themselves but must remain open for positive and negative consequences of competition as they appear from the perspective of different actors involved (Hölkeskamp, 2014; Jessen, 2014; Tauschek, 2013; Werron, 2015).

#### If the concept is normative, how is competition considered?

In economics, competition was historically linked to (political) liberalism and was, therefore, viewed as something beneficial. Adam Smith, for instance, argued that competition among producers prevents monopoly rents: “the price of monopoly is upon every occasion the highest which can be got. The natural price, or the price of free competition, on the contrary, is the lowest” (Smith, 1976 [1776]: 56). Hence, free competition benefits consumers and, in Smith’s understanding, society as a whole (see Werron, 2014b: 67–68). Similarly, Mill, when criticizing landlords and the gentry (notably in Mill, 2001 [1844]), viewed competition as a means to protect the weak (Mill, 1909 [1848]: 191). Yet, while he regularly stressed its benefits, he also acknowledged negative consequences for justice and social cohesion.

In the 20th century, Friedrich Hayek took a clear pro-market and pro-competition stance. He described competition as a “process of discovery,” uncovering “facts that would otherwise remain unknown or, at least, unused” (Hayek, 1969: 249; translation CGR). Ordoliberal economists such as Eucken (2004 [1952]) and Feld (2000) followed his footsteps, upholding competition as a politically preferable normative principle. Understanding these contributions requires a consideration of their normative vantage points, especially since they refer to—allegedly—descriptive results such as the two welfare theorems to justify further liberalization processes (e.g., Feld, 2000; Vanberg, 2001).

Theoretical accounts supporting competition also exist outside economics. Simmel, for instance, argues that competition defines modernity and allows resolving social struggles amidst scarcity without direct conflict. Moreover, since competitors attain scarce goods through high performance as determined by a third party, it can enhance overall welfare when these criteria align with broader social values (Simmel, 1995 [1903]: 225). Furthermore, Simmel also sees an integrating and socializing effect as competitors need to develop an understanding of the intentions and of the values held by the third party distributing the scarce good (see also Gane, 2019; Simmel, 1995 [1903]: 227).

Others were more critical of competition. French utopian socialists like Fourier and Sismondi highlighted its negative consequences for workers and French cities in the early 19th century (Fourier, 1996 [1808]). Later, Friedrich Engels, through empirical studies among the working class in England, highlighted the negative effects of competition by leading to a “battle of all against all” (Engels, 1969 [1891]: 73), and Karl Marx linked the competitive logic alongside technological progress with objectification and alienation (Marx, 1959 [1844]).

Several sociological and anthropological accounts of competition that, although acknowledging positive effects of competition in the era of modernity, see its rising importance and changing forms as problematic. Scholars like Nullmeier and Rosa stress that recent forms of competition create insecurity and a pressure for individual performance (Davies, 2017; Nullmeier, 2002: 172; Rosa, 2006). In the context of neoliberal ideologies, they argue that competition has shifted from a means to an end in itself (Nullmeier, 2002: 173; Rosa, 2006: 94–95). Rosa therefore calls for a restriction of competition in various social fields (Rosa, 2006: 102–104), a view echoed by sociologists like Wetzel (2013: 270) who criticize the formative role of competition as comprehensive governing principle of human conduct.

## Two additional and more general take-aways

The previous Section illustrated how the framework can be used on a theoretical level.^9^ Here, we want to specify how it can facilitate a joint meta-theoretical understanding of competition in the context of interdisciplinary research and suggest how applied researchers can use it to embed their empirical work into a consistent theoretical framework. Thereby, this Section delineates two more general conclusions regarding interdisciplinary competition research: first, using the framework reveals that differences *between* disciplines are not necessarily more pronounced than differences *within* disciplines. Second, the framework suggests that comprehensively understanding modern competition benefits from triangulating theories and tools from multiple disciplines. Here, the framework can both help identifying the right theoretical framework for the case at hand and (b) map the concepts you find in empirical research to academic concepts.

### Relevant differences and commonalities exist both within and between disciplines

The general definition from the second Section embraces a wide variety of situations, which are analyzed by scholars from distinct disciplines. In this context, the proposed framework can help understanding differences and commonalities *across* as well as *within* disciplines.

In some areas of the framework, differences between disciplines are clear. For instance, while large parts of economics focus on situations in which the scarce good gets (or could be) distributed via a price mechanism, the focus in social and cultural sciences is often on the institutionalization of competition, on the relevant criteria, and on its social implications, often sidelining the price mechanism.

In other respects, the differences between disciplines do not appear to be so crucial, and there are substantial differences in competition research *within* the same discipline, as well as commonalities *across* disciplines. Regarding the *scope* of competition, for instance, the seemingly clear focus of political economy on competition for commodified goods and of sociology focusing on competition for non-commodified goods is contradicted by early economic sociology and substantivist economic anthropology, which sought to understand the logic by which commodified goods are distributed in different societies. The concept of competition becomes even more blurred when we consider universal interpretations relevant to both sociology (Bourdieu) and economics (Becker).

A similar situation presents itself when it comes to the dimension of *methodology*: while it first appears that economists have a clear preference for model-based theorizing, and social and cultural studies prefer abstract-direct analysis (focused on concrete cases), many economists outside the mainstream criticize this mainstream obsession with models. At the same time, in social and cultural studies some theoretical approaches, such as Georg Simmel’s concept of competition or contributions from analytical sociology, are characterized by model-like features.

The same is true for the *normative orientation* of competition theories: Declaratively descriptive approaches exist in economics as well as in social and cultural sciences. While normative approaches in economics tend to highlight advantages of competition and social sciences often criticize its social implications, there are exceptions to this trend, like Georg Simmel’s account of competition as a peaceful fight.

In all, going through the different elements of the framework for different disciplines reveals that while there are differences *between* disciplines, there are also considerable differences *within* disciplines. At times, there are surprising commonalities across disciplines. For instance, qualitative sociologists and evolutionary-institutional economists share more epistemological and methodological convictions than institutional and neoclassical economists. This applies to all three dimensions, that is scope, methodology, and normative connotation, indicating that the framework can benefit both interdisciplinary and disciplinary work, especially if one wishes to triangulate different paradigms from the same discipline.

### Benefits from triangulating concepts from different disciplines for empirical analysis

The proposed framework can also be utilized for empirical research in two ways. First, it can help connect theoretical concepts more transparently to empirical cases. While this might seem trivial for typical cases of competition, in practice one may observe a migration and sometimes mingling of different forms of competition across fields. For instance, when market-like competition penetrates non-economic realms as in the case of university rankings, or when organized competitions like sustainability ratings emerge in economic contexts (Stark 2020a, 2020b: 5). This aggravates the task of finding the right theoretical basis. In such cases, while an interdisciplinary approach often becomes a necessity, picking the right concept becomes tedious. In such situations, our framework proposed above can facilitate a systematic approach to choose (or amend or combine) the right theories to describe the competition considered in a more transparent manner.

Second, concrete realizations of competition in the social world might draw from ideas linked to concepts of competition that were developed for competition in other fields. For instance, when it comes to the introduction of competition in new contexts, such as in the allocation of building grounds for the construction of subsidized housing in Vienna, normative views of competition that are mainly related to economic competition, such as the idea of an enhancement of quality and innovation, might be brought forward. At the same time, the institutionalized form of competition proposed in this case explicitly opposes market competition by replacing price mechanisms in the allocation of building grounds through other criteria (Azevedo et al., 2024). So, different and seemingly contradictory views of competition or, rather, of different forms of competition coincide in this case. The proposed framework can help to raise awareness for and to better understand the resulting contradictions.

## Outlook

This paper is a first attempt to facilitate an interdisciplinary analysis of competition by providing a framework for a comparative analysis of concepts of competition across disciplines. Such an approach appears desirable because of the proliferation of the various forms of competition in different social contexts, as well as the diversity of conceptualizations of competition in academic discussions. Against this backdrop, the framework compares different conceptualizations of competition regarding their scope, methodological orientation, and normative connotation. We applied it to selected contributions of influential scholars across disciplines to illustrate its potential to reveal commonalities and differences across and within disciplines, and also discussed its value for empirical competition research. Here, the framework can also help to discern cases that warrant an interdisciplinary approach because of relations between contexts from situations that are so different that they are best left to disciplinary studies. In all, we hope this work may facilitate a more nuanced understanding of processes of competitization (e.g., Wolfmayr, 2024).

Despite its preliminary results, the present endeavor necessarily remains incomplete in several ways: first, although intended as an interdisciplinary contribution, most authors discussed come from economics, sociology, political science, and anthropology. Future research might complement this contribution by applying the framework to further disciplines, such as business studies, philosophy, social psychology, or biology. Likewise, our focus on influential theories of competition has created a gender and ethnic bias, which could be addressed explicitly by future applications.

Second, we did not address the instrumentalization of competition within broader ideological movements, like fascism that justifies racist policies by referring to competition as a means to ensure the “survival of the fittest.” Such interpretations have a long-standing academic history that could be explored in future research. Third, we primarily examined the dimensions of scope, methodology, and normativity in isolation. Future work might explore their interdependencies: a scholar’s pre-analytic vision could shape her normative view on competition making her more comfortable using research methods such as simple general equilibrium models that tend to associate more competition with better outcomes. Alternatively, working regularly with methods where the default effect of competition is positive might shape a researchers’ normative view on competition.

Fourth, we did not discuss policy implications and the resulting interdependencies. Many economic theories suggest a positive effect of competition, notably perfect competition. If one believes these theories, it makes sense to establish institutions that protect or foster competition, like the Bureau of Competition in the U.S. These policies might extend beyond economics to reconfigure welfare institutions or education systems, enhancing national competitiveness—a topic widely debated in the “competition state” literature (Cerny, 2010; Sum, 2015). This change alters the institutions governing competitors and potentially the competition’s scope. This suggests a dynamically changing interdependency between scope, methodology, normativity, and policy actions. Studying these interdependencies and linking them with economic model performativity promise intriguing results.

Finally, we presented the framework as a tool for interdisciplinary research, only noting its potential usefulness for facilitating disciplinary research seeking to triangulate research programs from within one discipline. But one could further ask: when might it be more practical to “only” modify disciplinary research without engaging in interdisciplinary research at all? This question is intriguing as interdisciplinarity is often cherished publicly, yet most academic institutions still favor disciplinary research. Thus, there is a pragmatic case for altering disciplinary research, especially as interdisciplinary work might not be feasible for scholars facing institutional pressure to excel according to disciplinary criteria.

Notwithstanding this inevitable incompleteness, we hope that we have succeeded in making a constructive proposal for consolidating work on competition across disciplines and, thereby, in facilitating a genuinely interdisciplinary investigation of the concept.

## Notes

1. Competition is a relevant subject in even more disciplines, above all, business studies. Yet, taking them into adequate consideration is simply beyond the scope of the present paper, and their relevance can be discussed only briefly in the outlook.

2. This assessment is shared, for example, by Arora-Jonsson et al. (2021a: 5–12) and Werron (2015: 186). At the same time, it is important to note that, historically, there are multiple entanglements between disciplines, and also today, there have been endeavors to cross disciplinary boundaries. These will be discussed in the fourth and fifth Section. Nevertheless, we feel that the majority of work is—on a theoretical, topical, or methodological level—mainly disciplinary.

3. This means that we see the main area of application for our framework in cases where either scholars have already decided to take an interdisciplinary view or whether they are seriously considering it. While the framework could also be of interest for scholars working entirely within their disciplines (especially for the reasons discussed in the fifth Secion), this is not the main target audience of this work.

4. The main focus of this paper, however, will be on illustrating possible applications to sort out differences between theoretical positions. Its potential for empirical applications can, for reasons of space, be illustrated very concisely in ultimate two Sections and must mainly remain a topic for future applications.

5. Game theory as a particular method has been so successful in the context of bringing together different disciplines that one might even call it an interdisciplinary framework itself. Yet, it differs from the framework discussed here in the sense that game theory is a particular method that falls into the category of model-based analysis. In this sense, it is much more applied that the meta-framework introduced in this Section. If we consider the other dimensions—scope and normativity—game theoretical analysis can cover all of the cases discussed there.

6. The classical example is the construction of the periodic table in chemistry by Dmitri Mendeleev. The extent to which Weisberg’s distinction is also applicable within cultural and social studies has not been discussed extensively and should be subject to further investigation. We are, nevertheless, convinced that this distinction helps to explicate methodological differences between concepts of competition.

7. In the US-American system, the distinction between “international relations” and “political science” emerged, with the latter being more concerned with national and sub-national, and the first with international political processes. Not surprisingly, theories of competition from the two fields had the corresponding foci. Outside the US, both fields usually fall under to name “political science.”

8. Also, this does not mean that economists have analyzed competition exclusively through models; paradigms such as original institutionalism or the Regulation School have been very skeptical of the use of models and focused on what Weisberg (2007) describes as abstract-direct analysis (see already Wilber and Harrison, 1978). Also, there are important differences between the kind of models used in different paradigms (e.g., the maximization-cum-equilibrium models in the mainstream vs the disequilibrium models based on bounded rationality in evolutionary economics).

9. For reasons of space and readability, this paper does not contain an exemplary application of the framework in the context of empirical work. For such applications, see, for example, Pühringer et al. (2021) or Altreiter et al. (2024).
